# Extended mechanical loads for the analysis of acetabular cages

**DOI:** 10.1007/s10237-023-01728-z

**Published:** 2023-06-02

**Authors:** Martin O. Dóczi, Róbert Sződy, Péter T. Zwierczyk

**Affiliations:** 1 Department of Machine and Product Design, Faculty of Mechanical Engineering, Budapest University of Technology and Economics, Műegyetem rkp. 3., Budapest, H-1111 Hungary; 2 Dr. Manninger Jenő Trauma Center, Fiumei út 17., Budapest, H-1081 Hungary

**Keywords:** Hip joint loads, Finite element model, Acetabular cage, Orthopaedics

## Abstract

To analyse the strength and mechanical behaviour of hip implants, it is essential to employ an appropriate loading model. Generating computational models supplemented with muscle forces is a complicated task, especially in the initial phase of implant development. This research aims to expand the possibilities of the simpler acetabular cage model based on joint loads without significantly increasing the demand for computing resources. A Python script covered and grouped the loads from daily activities. The ten calculated major loads were compared with the maximum of the walking and stair climbing loads through the finite element analyses of a custom-made acetabular cage. Sensitivity analyses were performed for the surrounding bones’ elastic modulus and the pelvis boundary conditions. The major loads can geometrically cover the entire load spectrum of daily activities. The effect of many high-magnitude force vectors is uncertain in the approach that uses the most common maximum loads. Using these resultant major loads, a new stress concentration area could be detected on the acetabular cage, besides the stress concentration areas induced by the loads reported in the literature. The qualitative correctness of the results is also supported by a control computed tomography scan: a fracture occurred in an extensive, high-stress zone. The results are not sensitive to changes in the elastic modulus of the surrounding bone and the boundary conditions of the model. The presented load vectors and the algorithm make more extensive static analyses possible with little computational overhead. The proposed method can be used for checking the static strength of similar implants.

## Introduction

The hip joint is a spherical joint. The joint loads pass through the centre of the spherical head of the femur or through an artificial ball, which represents the head of the thigh bone when hip arthroplasty is required. When a large acetabular bone defect is presented, patient-specific solutions, such as custom-made implants, are mandatory. In such cases, geometric reconstruction and mechanical analysis are challenging problems (Ahmad and Schwarzkopf 2015; Paprosky et al. 1994).

To develop these types of implants, finite element (FE) analyses are used, among other methods. The FE models around the pelvis have evolved significantly over the years in terms of the material and the boundary conditions-loading models.

Bergmann et al. (2001) suggest that for biomechanical and FE analyses, the maximum load during normal walk and the maximum load while walking upstairs and downstairs should be considered.

Many publications have used joint forces for the loading model, or the suggested maximum load of the gait cycle, or sometimes the maximum load from ascending stairs (Costin et al. 2014; Plessers and Mau 2016; Totoribe et al. 2018; Vogel et al. 2020). In one publication, the data were supplemented with loads for stumbling (La Rosa et al. 2016). The maximum normal walking load in a different patient-specific model was investigated in a previous publication (Dóczi et al. 2020), and the results were consistent with the observed implant failure. Another publication preferred to use a model standing on one leg (Kawanabe et al. 2011), which others used but with hemipelvic boundary conditions (Moussa et al. 2020). The use of vertical loads of different magnitudes was also discussed by several authors (Du et al 2020; Fu et al. 2018; Iqbal et al 2017; Maslov et al. 2019).

However, if an implant can withstand a large load from a certain direction, it is not evident whether it would be strong enough to resist a smaller load from a completely different direction. Separate loads sampled from certain phases of the walking load are presented in Ma et al. (2013). Typically, five loads are modelled, for which individual analyses were carried out (Hsu et al. 2007; Maslov et al. 2021), but a 32-load model also exists (Wang et al. 2017), which is highly detailed, and examines the contact conditions of the acetabulum. One publication examines the importance of different load directions by considering five load cases (the maxima of one-leg stance, normal walking, ascending and descending stairs, and stumbling) (Del-Valle-Mojica et al. 2019). This concept is important for topology-optimized implants, because if some loads were not intended in the optimization task, it could provide poorly optimized results. Iqbal et al. (2019) used multiple load cases for their topology optimizations. Still, these were only the maximum values of the following daily activities: normal walking, standing on a single leg, standing up, sitting down, ascending stairs, and descending stair.

The hypothesis of our study is that these most common loads from the literature (the maximum load of walking and the loads of walking upstairs and downstairs) can only provide a narrow segment of sample for analysing acetabular implants. This will be supported by comparing these load magnitudes and directions with the force vectors from daily activities and with a patient-specific finite element model of a custom-made acetabular cage, where the stress concentration areas will be investigated.

Sensitivity analyses will be carried out for the material model and the boundary conditions. Moreover, the von Mises stress field will be compared with the observed implant failure from the patient’s control computed tomography (CT) data. The other goal of this study is to propose a heuristic algorithm for calculating the recommended loads for implants from any given dataset, albeit with some restrictions.

## Materials and methods

### Covering vectors

Calculating the mechanical response of a structure when its load vectors have the same initial point in every different load case would be computationally very challenging. A more effective way would be to groups the loads and define the so-called major loads, which may be used for simulations, where there are no other larger loads in their small surrounding areas.

**Fig. 1:**
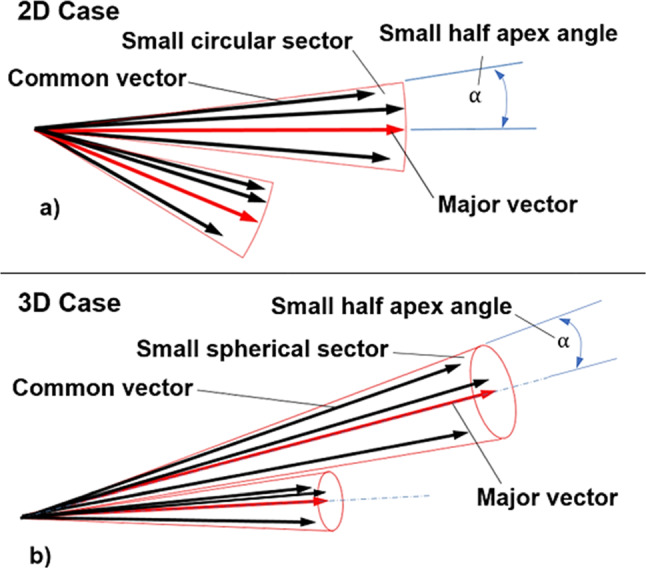
Major vector explanation in 2D (**a**) and in 3D (**b**)

The small surrounding area can be defined as a circular sector in the 2D case; in 3D, it is a spherical sector (see Fig. 1).

The apex angle is user-defined. With a smaller angle, the calculation is more detailed, but this can increase the number of simulations required. If the apex angle is large, the coverage area is larger, and fewer major loads are required. However, this method can produce poor simulation results because the direction difference between the major vector and a common vector can be significant.

Calculating the major loads is similar to the minimal disc covering problem, which is non-polynomial (NP) hard (Das et al. 2011). In our case, the task is to cover 3D vectors with spherical sectors with a given half-apex angle, which the authors selected at 10°. The motivation for this decision will be explained later. In this study, a possible heuristic solution will also be provided.

### Heuristic algorithm for calculating the major loads

The dataset of the loads was the HIP98 dataset by Bergmann et al (2001). All the given loads from daily activities (slow, normal, and fast walking, walking upstairs, walking downstairs, standing up, sitting down, standing, knee bending) for an average patient were considered. These data contain 9 × 201 = 1809 3D vectors with their X, Y, and Z components. Our study focused on loads of the acetabular joint in the pelvic coordinate system (Bergmann et al. 2001).

The loads have the same initial point, which is their origin. Thus, the load vectors are represented as their terminal points on the figures.

**Fig. 2:**
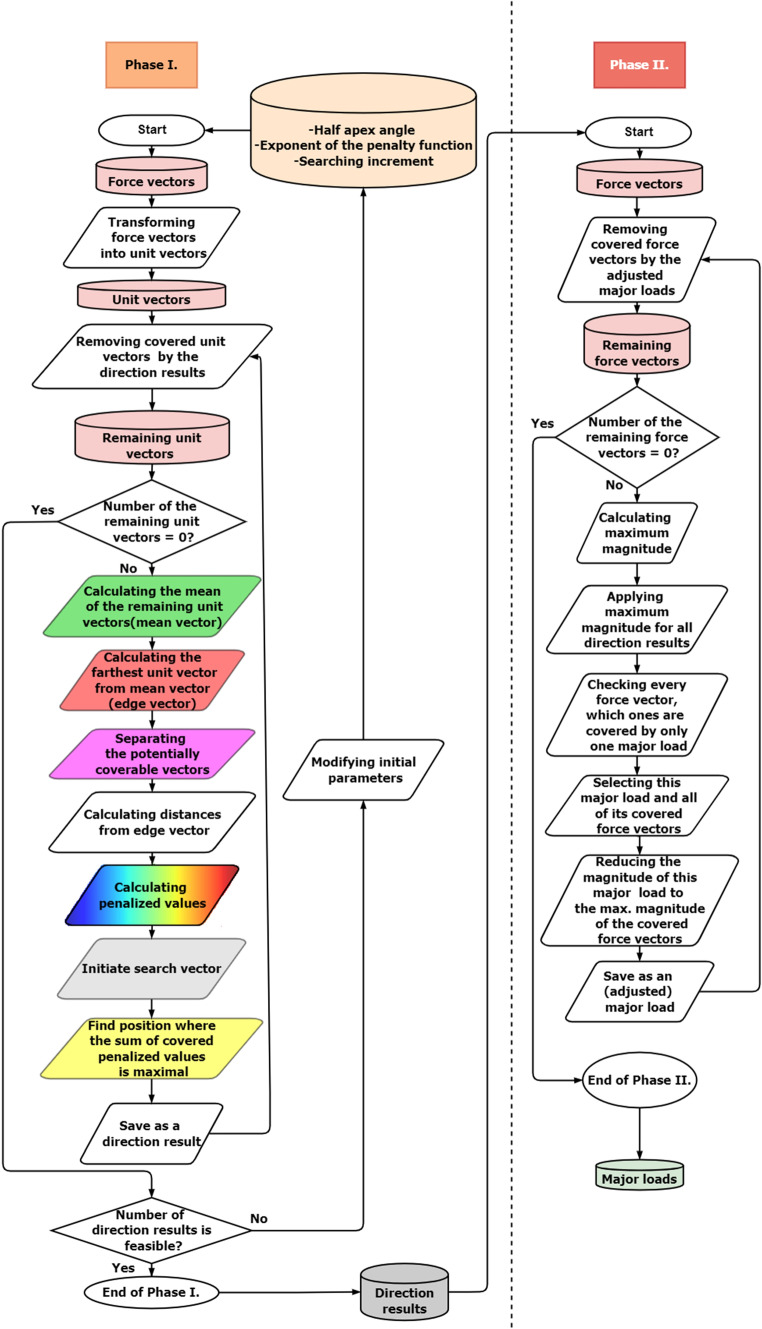
The flowchart for the calculation of the major loads direction and magnitude

The heuristic algorithm was written in Python and can be divided into two parts (see flowchart in Fig. 2).

**Fig. 3:**
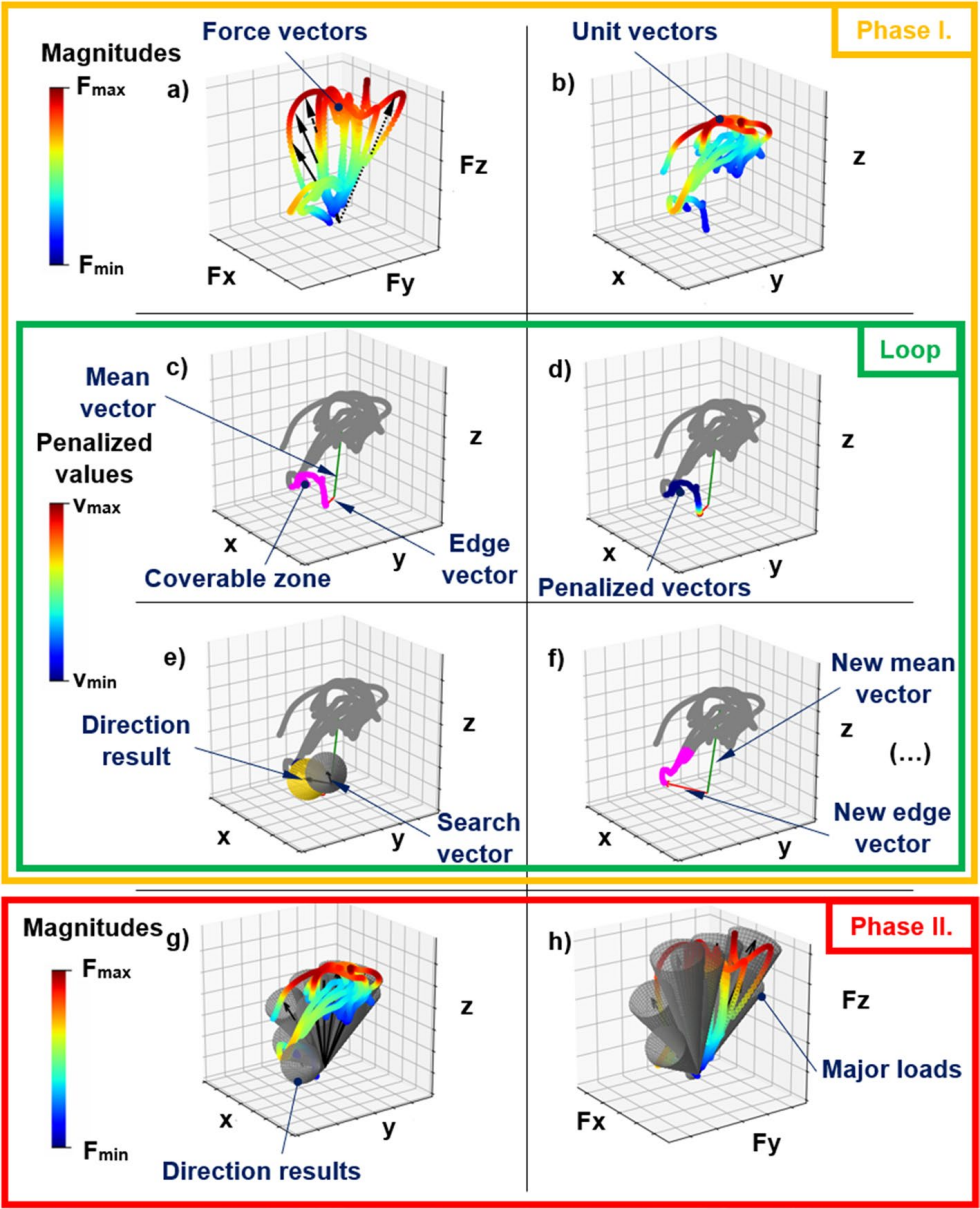
The endpoints of the force vectors are coloured by their magnitudes, some of them are marked with black arrows (**a**); transformation to unit vectors (**b**); calculating the mean and the edge vector, separating the possible coverable unit vectors (**c**); penalizing the coverable unit vectors depending on their distance from the edge vector (**d**); the search vector in its initial position (grey) and local best coverage (gold) (**e**); the subprocess repeated, previously covered unit vectors were removed (**f**); the direction results (**g**); the length-adjusted direction results, the major loads (**h**)

The images in Fig. 3 also demonstrate the process, using colour markings similar to Fig. 2.

In the first part, to calculate the directions of the major loads, the data’s magnitudes are irrelevant, so the force vectors should be transformed into unit vectors.

The next step is to calculate the mean of this unit vector data; then, the algorithm calculates the farthest data point from this global mean vector. This edge vector can be applied to separate the local set of the unit vectors to reduce computing time.

In the following step, the search vector was defined. The search vector is the axis of a cone, which has a user-defined half-apex angle ($\vartheta$ = 10°). If this covers a vector, the following equation is satisfied [Eq. (1)]:

1 $$\left( {{\varvec{u}} \cdot {\varvec{c}}} \right)^{2} - \left( {{\varvec{c}} \cdot {\varvec{c}}} \right) \cdot \left( {{\varvec{u}} \cdot {\varvec{u}}} \right) \cdot \left( {\cos \vartheta } \right)^{2} \ge 0$$

where ***u*** is the vector be covered, ***c*** is the rotation axis of the cone as a vector, and $\vartheta$ is the cone’s half-apex angle.

The search vector can also move around the edge vector, but it always contains the edge vector. The increment of the movement is user controlled. The increments define the number of different search positions (the search resolution) and the details of the calculation, including running time.

The next step is to choose an appropriate position from among these various positions: this will be defined by the search vector, and it will be the direction of a major load. Fewer major loads are produced if the algorithm is set to prefer to cover the unit vectors closer to the edge vector. This can be implemented as described below.

The distances from the edge vector to every local unit vector are calculated. Next, all the distances are normalized by the longest distance (*d*_norm_). After that, the following penalty function is used [Eq. (2)]:

2 $$v = v_{{{\text{min}}}} + \left( {1 - v_{{{\text{min}}}} } \right) \cdot \left( {1 - d_{{{\text{norm}}}} } \right)^{p}$$

Hence, all unit vectors have a certain value, (*v*). Each unit vector has a minimum value (*v*_min_), and if the exponent (*p*) is greater than 0, the closer the unit vector is to the edge vector, the higher the *v* value will be.

The sum of these values is calculated in every search vector position. Where it is the maximum, that will be the direction of a major load, and the local unit vectors covered by this major load’s cone are removed.

The process is then repeated in a loop to calculate the new mean values of the remaining unit vectors until all the unit vectors have been covered.

In summary, the user-defined values were the following. It was possible to cover the dataset with ten major loads, choosing the exponent of the penalty *p* = 6, the half-apex angle $\vartheta$ = 10°, and the increment of the movement was 1°.

**Fig. 4:**
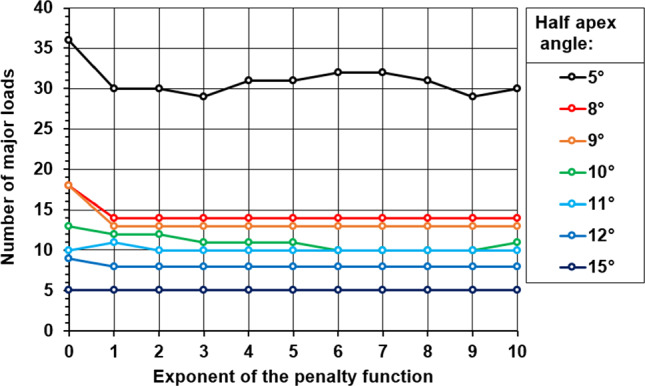
Penalty function exponent and half-apex angle impact on major load vector count

Figure 4 shows the chosen parameters. By modifying the exponent of the penalty function, different numbers of major load vectors can be used to cover the dataset. In the case of a half-apex angle of 10°, this number can be reduced to 10 (by setting the exponent of the penalty function to 6), which is comparable to the number of types of loads from daily activities in the data set (10 vs. 9). It was not advisable to run more simulations than this to demonstrate the method.

Selecting a 1° increment of the movement, the running time for calculating the major loads was feasible. Smaller increments did not significantly improve the algorithm’s performance in terms of fewer major loads but increased the running time significantly.

The second part of the algorithm calculates the magnitudes of major loads. First, all the major load direction magnitudes are changed to the maximum magnitude from the dataset of the force vector, and these are then reduced step by step as described in the process above (Fig. 2).

### Finite element model of the fixation

To examine the applicability of the major loads, a patient-specific finite element model was developed.

The patient had a large Paprosky 3/b acetabular bone defect. The treatment was a custom-made sheet metal acetabular cage provided by Sződy et al. (2017). The CAD models were created using the patient’s anonymized pre- and postoperative CT data. The hemipelvis, the acetabular cage and its screws were segmented with threshold-based and manual segmentation using Slicer 3D. Then, the STL files were exported and repaired with Autodesk Meshmixer.

To produce the solid CAD model of the pelvis, SolidWorks 2018 Scanto3D module was used. Near the bone defect, the cortical and the trabecular bone cannot be segmented easily. Due to the migration of the original acetabular component, a significant change had taken place in the bone’s structure. Therefore, the regions close to the acetabular defect were separated from the other parts of the pelvis, and different materials were used for the models, which will be discussed later.

The mid-surface CAD geometry of the sheet metal acetabular cage was also made in SolidWorks 2018.

The screws had a simplified cylindrical geometry, with a nominal diameter of 4.5 mm. The heads of the screws had simplified spherical geometry with dimensions matching the relevant standard (ISO 5835:^1991^). The liner and the femoral ball were modelled as revolved bodies.

**Fig. 5:**
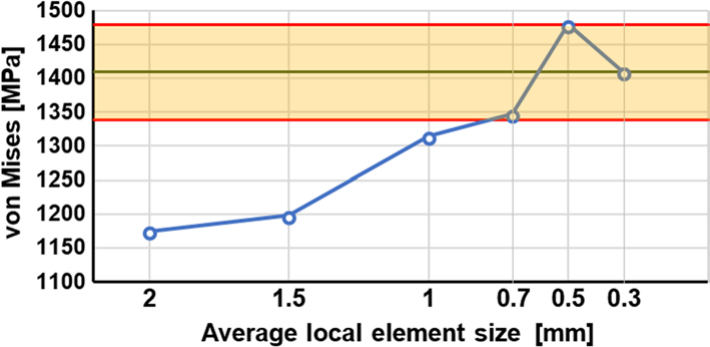
Mesh refinement analysis for the acetabular cage

The pre-processing of the finite element model was carried out using HyperMesh 2017.2. Multiple mesh refinement was applied to the acetabular cage because this part is the major focus of this study. The maximum von Mises stress was evaluated in a stress concentration area with different element sizes for the normal walking load case. Between 0.3 and 0.7 mm element sizes, the deviation between the results was under 5% compared to the last mesh refinement (Fig. 5).

This element size range was used in all stress concentration areas. The final FE mesh data are given in Table 1.

**Table 1:** The FE mesh data

Part	Element type	Number of elements	Thickness of shell elements
Acetabular cage	8-node second order quad	11,925	1.5 mm
Cortical bone	6-node second order tria	15,961	1 mm
Other solid parts	10-node second order tetrahedron	159,587	–

Homogenous, linear elastic, and isotropic material properties were used everywhere, including the bone (Anderson et al. 2005). Near the acetabular defect, a homogenous material was used with an averaged Young’s modulus (Ravera et al. 2015). A sensitivity test was performed for this part due to the versatile bone structure.

In the healthy areas of the pelvis, a separate material model was applied. The solid elements represented the spongious part of the pelvic bone, while shell elements represented the cortical layer (Anderson et al. 2005; Plessers and Mau 2016), as shown in Table 2.

**Table 2:** Material properties

Material	Young’s modulus (MPa)	Poisson’s ratio
Steel (AISI 316L)	192,000	0.3
Liner (cross-linked polyethylene)	1000	0.4
Cortical bone	17,000	0.3
Trabecular bone	200	0.3
Homogenous bone	7 000	0.3

Table 3 shows the connections between the parts of the model.

**Table 3:** Connections between the model parts

Connection between	Interface type	Frictional coefficient	Comment
Thread-bone	Bonded	–	–
Liner-cage	Bonded	–	–
Cage-bone	Frictional contact	0.3	Chang et al. (2014)
Cage-Screw’s head	Frictional contact	0.23	Chang et al. (2014)
Liner-ball	Frictional contact	0.02	For numerical stability

There were fixed boundary conditions at the sacroiliac joint and the pubic symphysis (Clarke et al. 2016; Plessers and Mau 2016). A sensitivity analysis was performed on the boundary condition change in the pubic symphysis.

The first load step is a bolt pretension, where every bolt has a 50 N pretension force, modelling the surgical procedure and closing the contacts. Then, in the next load step, as the main load, a force vector is applied to the centre of the femoral head. Each load was applied in a separate static load case after the bolt pretension, as the aim of the study is to investigate the response of the cage to different load directions. The applied loads based on the literature and simulated by the algorithm can be seen in Table 4.

**Table 4:** The components of the main loads

Load name	*x* component (N)	*y* component (N)	*z* component (N)
Slow walking	141	− 122	1897
Normal walking	228	− 207	1803
Fast walking	292	− 377	1905
Walking upstairs	158	− 468	1912
Walking downstairs	338	327	1989
Major load 1	519	519	1910
Major load 2	362	− 579	1855
Major load 3	478	− 353	1840
Major load 4	278	94	1885
Major load 5	770	131	1502
Major load 6	321	− 953	1361
Major load 7	710	− 1006	848
Major load 8	543	− 488	940
Major load 9	499	− 143	728
Major load 10	274	− 304	182

The components of the main loads for FE analyses in the pelvic coordinate system (Bergmann et al. 2001) are given in Table 4. The first five rows are the peak loads from different daily activities from the HIP98 database (slow, normal, and fast walking; walking upstairs and downstairs) in BW% (percent of body weight). The patient’s weight was approximately 80 kg, and the force components were calculated with a 9.81 m/s^2^ acceleration of gravity. The next ten loads are the major loads that were given by the algorithm.

Small displacement quasi-static nonlinear analyses were performed because the deflections of the model were relatively small compared to its main dimensions, whereas the dynamic effect of the forces was neglected, and the frictional contacts required nonlinear calculation. The FE solver was Optistruct 2017.2.

The Python script used to determine the major loads and the FE calculations were run on a personal computer (Processor: Intel^®^ Core™ i7-9700 CPU @3.00 GHz; RAM: 64 GB; System type: 64-bits operative system, × 64-based processor).

The Python script for the major loads finished in 32 s using 199.6 MB of memory, while the 1 + 15 = 16 FE (15-load case after the pretension step) analyses were completed in 11.75 h.

**Fig. 6:**
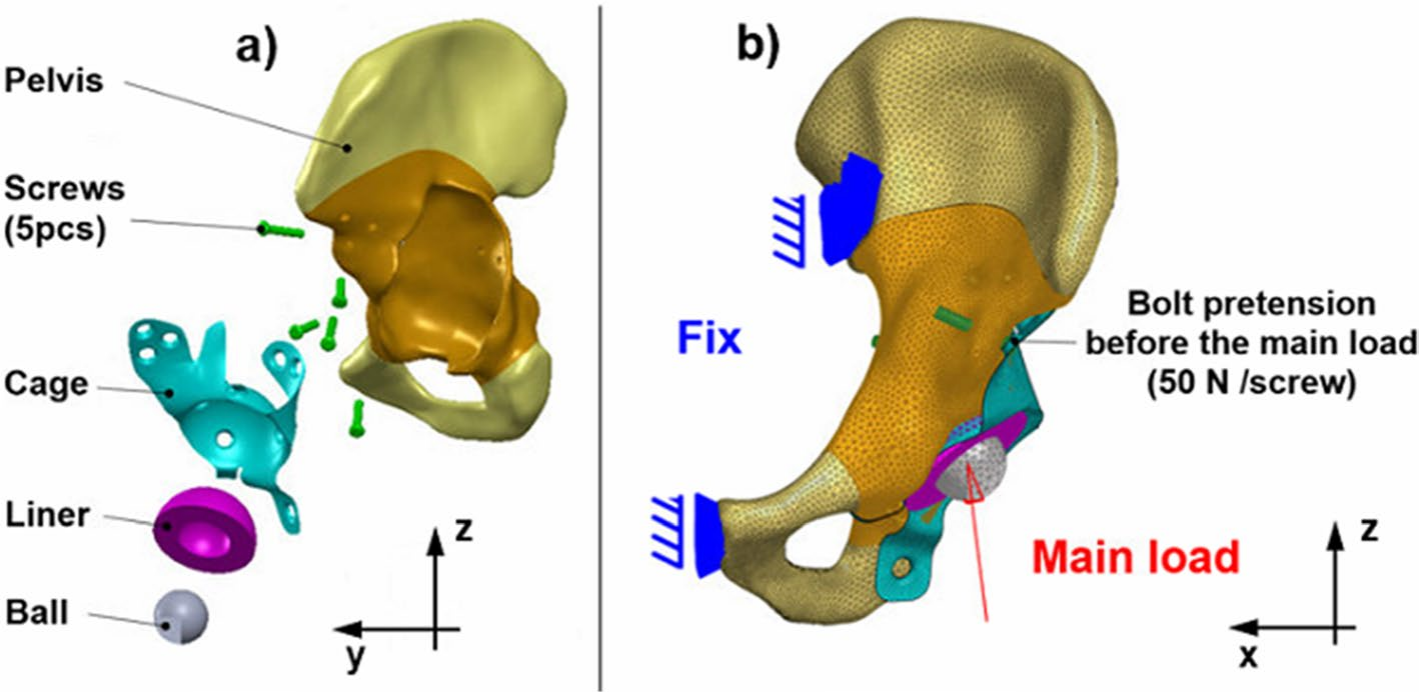
The geometry (**a**) and the finite element model (**b**)

The geometry and the FE model are depicted in Fig. 6.

The acetabular cage is the major focus of this study. The von Mises stress of the cage was examined during the sensitivity analyses in one load case. The preload was the bolt pretension, and subsequently, the maximum amplitude from the normal walking gait cycle was applied to the model as a static load. The sensitivity analyses were performed in two dimensions.

The homogenous bone part was in connection with the acetabular cage. Here, Young’s modulus value was changed by ± 30%, so the elastic moduli were 9100 and 4900 MPa.

The boundary condition at the pubic symphysis was changed to a 1 DOF constraint in the X-axis direction.

The patient’s postoperative and control CT scans were utilized for the qualitative verification of the results. The expected failure must be in an extensive, high-stress zone, which the FE model must be able to predict.

## Results and discussion

### Comparison of loads in the literature and calculated by the algorithm

**Fig. 7:**
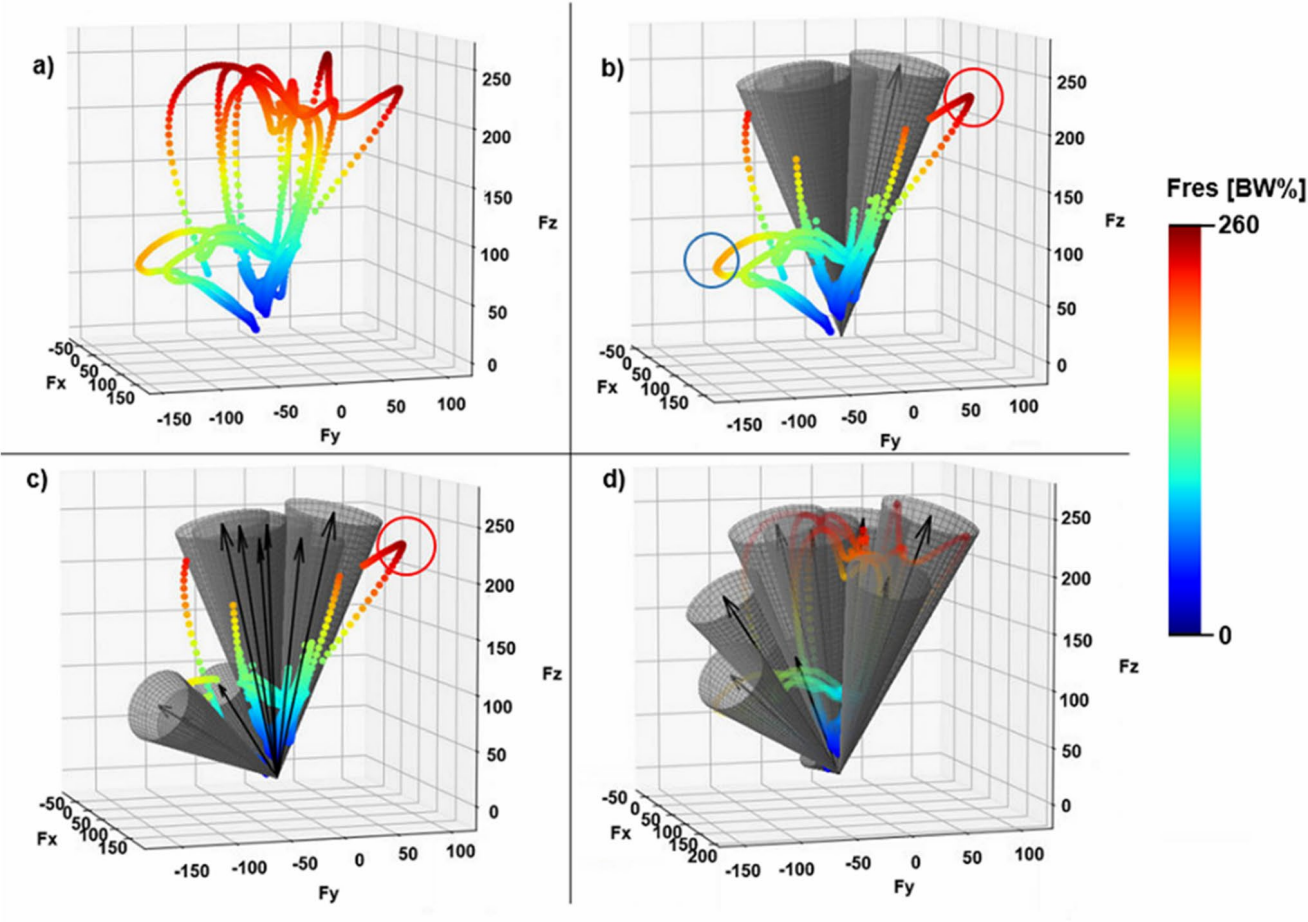
The dataset (**a**); covering with loads from the literature (Bergmann et al. 2001; Costin et al. 2014; Plessers and Mau 2016; Totoribe et al. 2018; Vogel et al. 2020) (**b**); covering with the peak loads of daily activities (**c**); covering with the ten major loads calculated by the algorithm (**d**)

Figure 7 compares the loads in the literature and the results presented by the algorithm. It clearly shows that a significant proportion of the loads from daily activities, including high-magnitude loads, are not taken into consideration. For example, there is a large vector with a 252 BW% magnitude (marked with a red circle), which is from the downstairs activity. In a quite different direction, there is a load with a large BW% magnitude (marked with a blue circle). An approach that uses the peak loads from the daily activities (i.e. nine loads) cannot cover all the force vectors. However, with these ten loads, our algorithm can cover all the force vectors from the daily activities with the spherical sectors. This method requires only one more FE analysis than when the maximum force approach was employed. FE analyses confirmed the fact that this extension influences the stress state of the cage.

### Finite element results

**Fig. 8:**
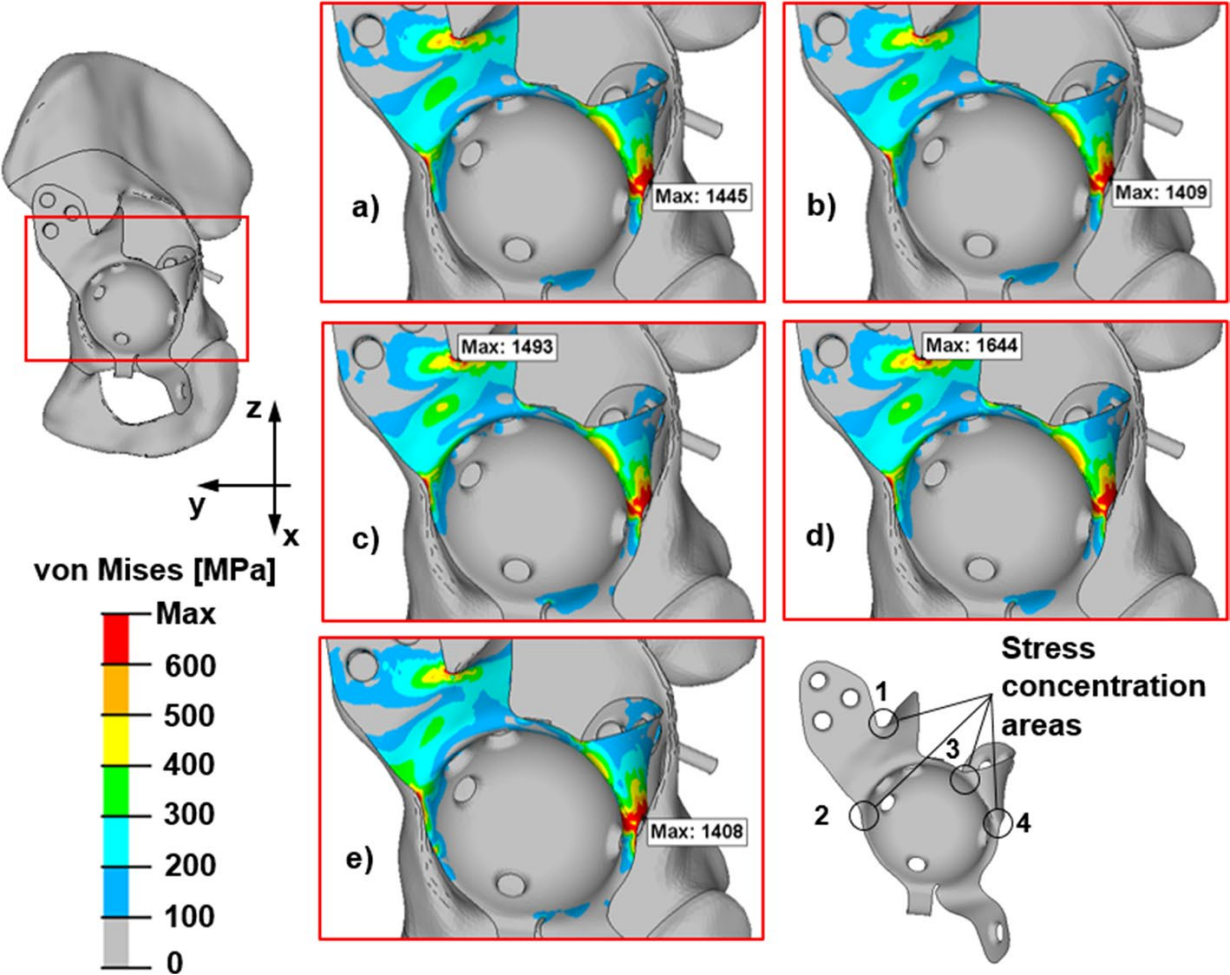
The stress concentration areas and the von Mises stress distributions for different peak loads produced by daily activities. Slow walking (**a**); normal walking (**b**); fast walking (**c**); ascending stairs (**d**); descending stairs (**e**)

Investigating the von Mises stress results and the loads given in the literature (Bergmann et al. 2001; Costin et al. 2014; Plessers and Mau 2016; Totoribe et al. 2018; Vogel et al 2020;), they are found to be very similar. Four main stress concentration areas can be detected (see Fig. 8). The shell elements have two layers, top and bottom. These figures show the von Mises stress on the layer with the greater stress. (The maximum von Mises stress will be presented.)

**Fig. 9:**
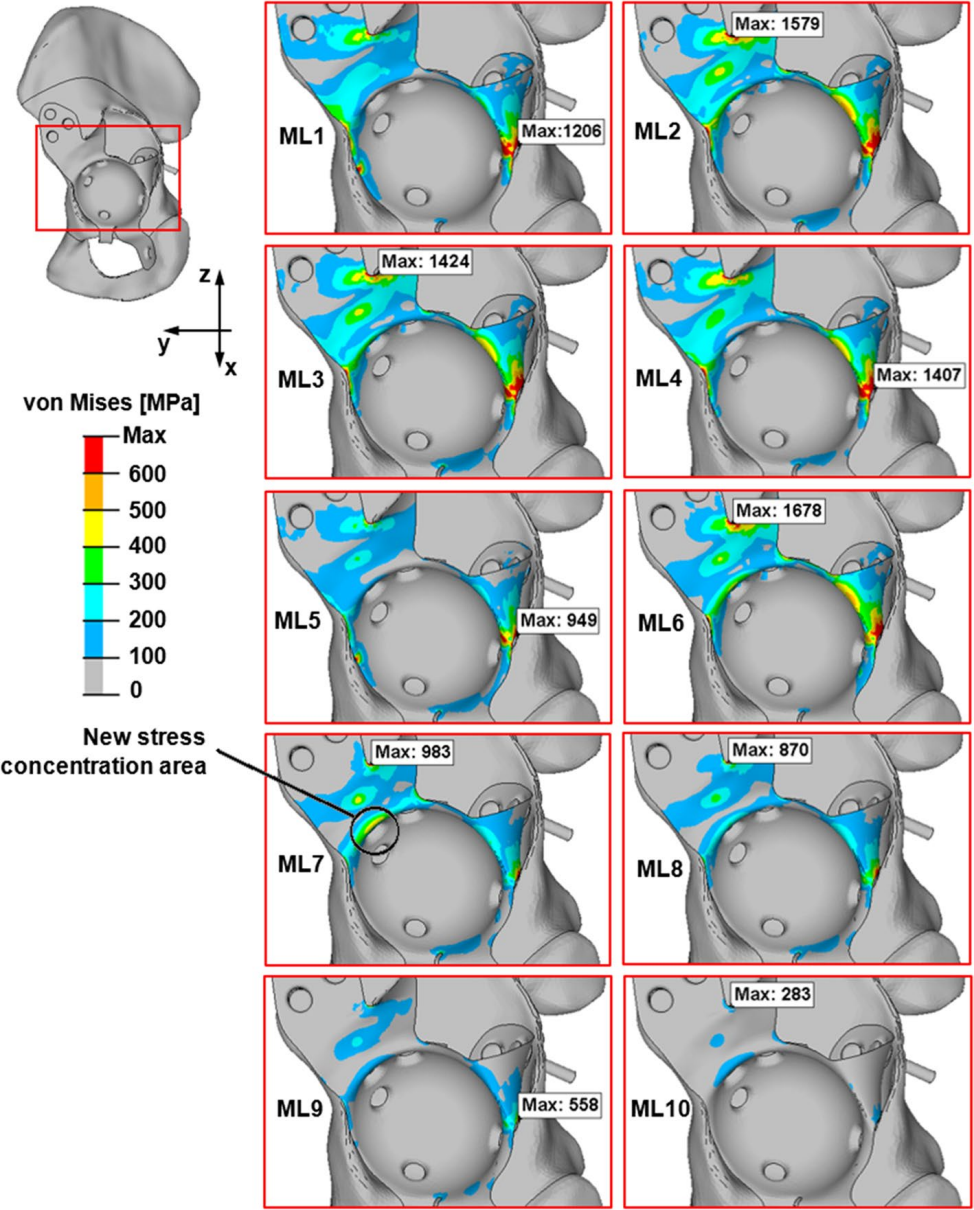
The von Mises stress distributions for the ten major load cases (abbreviated ML#)

Viewing the results induced by the major loads, a new stress concentration area, which the loads from the research of the literature cannot provide, can be detected, in addition to all the previously mentioned stress concentration areas (see Fig. 9).

### Sensitivity analyses

Overall, the examined stresses showed low sensitivity to the changes mentioned in Table 5. The acetabular cage’s nodal maximum von Mises stresses were compared to the original model. The change in the bone’s elastic modulus had no significant effect on the von Mises stresses of the acetabular cage because it is much stiffer than the bone and the supporting areas around it.

**Table 5:** The summarized results of the sensitivity analyses on the two layers of the cage

Changes	Layer	Maximum of the absolute differences in the von Mises stress field of the cage (MPa)	Mean of the absolute differences in the von Mises stress field of the cage (MPa)	Median of the absolute differences in the von Mises stress field of the cage (MPa)
Homogenous bone Young’s modulus value + 30%	Z1	35	2.64	1.39
	Z2	53.1	3	1.26
Homogenous bone Young’s modulus value − 30%	Z1	53.5	3.07	1.5
	Z2	66.4	3.46	1.31
1 DOF constraint at the pubic symphysis	Z1	15.8	1.04	0.54
	Z2	29.8	1.13	0.51

Different boundary conditions did not give significantly different results either; in both cases, almost the same kind of displacements was produced.

### Comparison of postoperative and control CT data

**Fig. 10:**
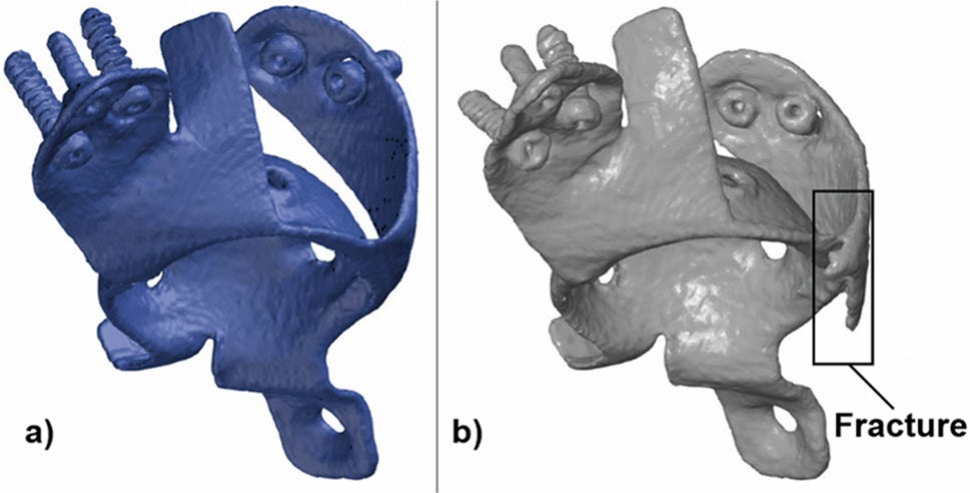
Fracture can be detected investigating the segmented STL files of the acetabular cage from the postoperative CT (**a**) and from the 1-year control CT (**b**)

The comparison of the STL meshes from the postoperative and control CT data can be seen in Fig. 10. The primary failure of this implant was a fracture in one of the supports, where two stress concentration areas (zone 3 and zone 4) were in a wide section with a high von Mises stress.

A fracture was detected in the control CT in this part of the cage. This failure was indicated by stress responses both to loads commonly found in the literature (the first five loads from Table 4) and stress responses to major loads modelled by our method. The authors want to emphasize that in this case, this type of qualitative verification is suitable for showing the consistency of the results from the loads from the literature with the results provided by the new method we propose.

### Limitations of the results

The major vector results are presented only for the 10° half-apex angle, and it is not proven that there is no coverage with less major load vector with this angle. Another limitation is that only the HIP98 database was covered because this contained the forces in the pelvic coordinate system.

The FE results only focus on the von Mises stresses of the cage. Moreover, the calculated von Mises stress results are greater than the yield stress of the unformed sheet metal. This is the consequence of using a linear elastic material model for the acetabular cage. The cage was a highly deformed sheet metal part. Due to the extensive cold work, the yield stress in the highly formed areas could be much higher than in the original. There was no annealing process after the forming so that residual stress could remain in the cage. However, modelling this phenomenon is challenging and has no other important effect on the result during the qualitative comparison of the stress concentration areas.

### Limitations of the study

The study and the methodology used also have their limitations, and some criticisms may be made of them, which we tried to reduce with countermeasures but which cannot be eliminated entirely.

The muscle loads cannot be included because the algorithm only deals with vectors with the same initial point. The major loads provided do not belong to any measured data point, and the other joint load is unknown. However, for implant development, it can produce viable results, and for topology optimization, simplified models are recommended.

The 10° half-apex angle selected was the author’s choice. A smaller angle could provide more detailed results, but it would mean more major loads and more simulations.

The heuristic algorithm cannot provide a globally optimal solution. It is worth running it multiple times with several parameters specified by the user. Nevertheless, it is guaranteed to provide a solution, and if the number of major loads is acceptable, it can be used immediately. Only static analyses can be performed with these forces.

Regarding the FE model, only a hemipelvis model without pelvic discontinuity is suitable for the analyses; the load on the other leg and the muscle forces and ligaments were replaced by boundary conditions. Accordingly, a sensitivity test was carried out for the boundary condition, but no significant change in the stress state of the cage was observed.

The starting point of the geometric reconstruction was a clinical CT scan with scattered X-ray photons. Segmentation, STL mesh formation, conversion into a CAD model, and finite element meshing again involve the possibility of geometric inaccuracies, which may arise due to the differences in the position and orientation of the acetabular cage in the model compared to in reality. To eliminate this potential error, we used screw pretensioning, but the pretension forces were also estimated, and the geometry of the screws was simplified. There may also be inaccuracies in the friction coefficients from the literature.

Based on the simplified calculation, a homogeneous, linear elastic, isotropic material model was used throughout. Where possible, separated material properties were used when specifying the material model of the pelvis, but the exact cortical thickness was only an estimate supported by the literature. Due to the limitations of the clinical CT data, it was impossible to separate the cortical and the spongiosis parts in the areas around the cage. To compensate for this uncertainty, a sensitivity test was performed on the modulus of elasticity of the parts here, which had no significant effect on the stress state of the acetabular cage either.

Elsewhere, other authors have been able to use the inclusion of muscular forces successfully (Clarke et al. 2016; Phillips et al. 2007; Ravera et al. 2015; Zaharie and Phillips 2018) and make sensitivity analyses to investigate the changes in the results. It is important to note that these publications primarily focused on the pelvis bone structure and the stresses around it. Considering all these muscular forces for simulations entails difficult and computationally expensive tasks, especially in the early phase of implant development.

Overall, these loads were able to predict the failure’s location, as was also seen from comparing the CT data. Hence, these results can be deemed to have been qualitatively verified. In this case, in terms of failure, the stress patterns given for the loads by the literature and those given by our proposed method match. Still, it is evident that further tests are needed to show the new stress concentration area, because the small sample size and the lack of a diverse range of implant designs and materials limit the study. Expanding the study to include a greater variety of implants would allow for a more comprehensive understanding of the stress concentration areas and potential failure locations. To achieve experimental quantitative verification, additional data are needed. Strain gauge stamp measurements could also confirm the stress concentration areas but that would require in vitro experiments.

## Conclusion

For the stress analyses of implants, the most common loads given in the literature can only provide a tight inspection segment with the FE method. During daily activity, large magnitude force vectors occur in quite different directions than the maximum peak loads of walking and stair climbing. Stress concentration areas may remain hidden if these loads alone are used for the investigation of implant stresses and the development of implants based thereon.

This paper proposes a new approach and method for the loading model of FE simulations of hip joint implants. This approach is mainly based on the statement that if many force vectors have the same initial point, the loads can be grouped by spherical sectors with small half-apex angles. The axes of these spherical sectors can be the direction of major loads. The magnitudes are the maximum magnitude of the covered vectors. Using the published heuristical method for an NP-hard covering problem, the major loads can be calculated quickly with quite good efficiency.

The other useful result from using the major loads is that it made it possible to select a zone for development because all the major loads predicted this zone as a stress concentration area (zone 4).

The methodology proposed here can be applied in concept formation, using simple models, in preliminary static checks. Subsequently, detailed calculations can be carried out for the promising product variants with more advanced models.

The algorithm can also cover other vectors with the same initial point, for example, the force vectors of a femoral implant.

In the algorithm, adding step zero could remove the force vectors that do not have a larger magnitude than a certain value. Suppose there is any previously given load with a specified half-apex angle (such as the stumbling with its great magnitude). In that case, the inner force vectors can also be removed, and the remaining vectors could be covered. This method and its results can be used for further implant analyses, even with patient-specific joint loads from inverse dynamics muscle models.

The FE model could be improved, for example, by including the applicable ligaments around the hemipelvis, a CT-based material assignment for the bone, and the modelling of the cage’s residual stresses during manufacturing, nonlinear material models and the detailed modelling of bone-screw connections, even with submodels. In addition, the authors will use the approach of major loads to study the topology optimization capabilities of acetabular cages.

## Data Availability

The external load vector data which were analysed in this study are available in *Data collection* *‘HIP98’* at https://orthoload.com/test-loads/data-collection-hip98/ (Bergmann et al. 2001). The medical image data are not openly available due to their sensitivity (human data).

## References

[CR1] Ahmad A, Schwarzkopf R (2015). Clinical evaluation and surgical options in acetabular reconstruction: a literature review. J Orthop.

[CR2] Anderson AE, Peters CL, Tuttle BD, Weiss JA (2005). Subject-specific finite element model of the pelvis: development, validation and sensitivity studies. J Biomech Eng.

[CR3] Bergmann G, Deuretzbacher G, Heller M, Graichen F, Rohlmann A, Strauss J, Duda G (2001). Hip contact forces and gait patterns from routine activities. J Biomech.

[CR4] Chang CW, Chen YN, Li CT, Peng YT, Chang CH (2014). Role of the compression screw in the dynamic hip–screw system: a finite-element study. Med Eng Phys.

[CR5] Clarke SG, Phillips ATM, Bull AMJ (2016). Evaluating a suitable level of model complexity for finite element analysis of the intact acetabulum. Comput Methods Biomech Biomed Eng.

[CR6] Costin S, Micu C, Cristea S, Dragomirescu C (2014). Process for realisation of a cage adapted to patient for specific acetabular defects in tha revision. UPB Sci Bull, Ser d: Mech Eng.

[CR7] Das GK, Fraser R, Lòpez-Ortiz A, Nickerson BG (2011) On the discrete unit disk cover problem. In: Katoh N, Kumar A (eds) WALCOM: algorithms and computation. WALCOM 2011. Lecture notes in computer science, vol 6552. Springer, Berlin, Heidelberg. 10.1007/978-3-642-19094-0_16

[CR8] Del-Valle-Mojica JF, Alonso-Rasgado T, Jimenez-Cruz D, Bailey CG, Board TN (2019). Effect of femoral head size, subject weight, and activity level on acetabular cement mantle stress following total hip arthroplasty. J Orthop Res.

[CR9] Dóczi M, Sződy R, Zwierczyk PT (2020) Failure analysis of a custom-made acetabular cage with finite element method. In: Steglich M, Mueller C, Neumann G, Walther M (eds) ECMS 2020. Proceedings of the 34th international ECMS conference on modelling and simulation. European Council for Modelling and Simulation, Wildau, pp 250–255. 10.7148/2020-0250

[CR10] Du Y, Fu J, Sun J, Zhang G, Chen J, Ni M, Zhou Y (2020). Acetabular bone defect in total hip arthroplasty for crowe II or III developmental dysplasia of the hip: a finite element study. Biomed Res Int.

[CR11] Fu J, Ni M, Chen J, Li X, Chai W, Hao L, Zhang G, Zhou Y (2018). Reconstruction of severe acetabular bone defect with 3D printed Ti6Al4V augment: a finite element study. Biomed Res Int.

[CR12] Hsu JT, Chang CH, Huang HL, Zobitz ME, Chen WP, Lai KA, An KN (2007). The number of screws, bone quality, and friction coefficient affect acetabular cup stability. Med Eng Phys.

[CR13] ISO 5835:1991 (1991) Implants for surgery—metal bone screws with hexagonal drive connection, spherical under-surface of head, asymmetrical thread—dimensions. International Organization for Standardization. https://www.iso.org/standard/12001.html. Accessed 29 May 2023

[CR14] Iqbal T, Shi L, Wang L, Liu Y, Li D, Qin M, Jin Z (2017). Development of finite element model for customized prostheses design for patient with pelvic bone tumor. Proc Inst Mech Eng H.

[CR15] Iqbal T, Wang L, Li D, Dong E, Fan H, Fu J, Hu C (2019). A general multi-objective topology optimization methodology developed for customized design of pelvic prostheses. Med Eng Phys.

[CR16] Kawanabe K, Akiyama H, Goto K, Maeno S, Nakamura T (2011). Load dispersion effects of acetabular reinforcement devices used in revision total hip arthroplasty: a simulation study using finite element analysis. J Arthroplasty.

[CR17] La Rosa G, Clienti C, Di Bella S, Rizza F (2016). Numerical analysis of a custom-made pelvic prosthesis. Procedia Struct Int.

[CR18] Ma W, Zhang X, Wang J, Zhang Q, Chen W, Zhang Y (2013). Optimized design for a novel acetabular component with three wings. A study of finite element analysis. J Surg Res.

[CR19] Maslov L, Surkova P, Maslova I, Solovev D, Zhmaylo M, Kovalenko A, Bilyk S (2019). Finite-element study of the customized implant for revision hip replacement. Vibroeng Proc.

[CR20] Maslov L, Borovkov A, Maslova I, Soloviev D, Zhmaylo M, Tarasenko F (2021). Finite element analysis of customized acetabular implant and bone after pelvic tumour resection throughout the gait cycle. Materials (basel).

[CR21] Moussa A, Rahman S, Xu M, Tanzer M, Pasini D (2020). Topology optimization of 3D-printed structurally porous cage for acetabular reinforcement in total hip arthroplasty. J Mech Behav Biomed Mater.

[CR22] Paprosky W, Perona P, Lawrence J (1994). Acetabular defect classification and surgical reconstruction in revision arthroplasty: a 6-year follow-up evaluation. J Arthroplasty.

[CR23] Phillips ATM, Pankaj P, Howie CR, Usmani AS, Simpson AHRW (2007). Finite element modelling of the pelvis: inclusion of muscular and ligamentous boundary conditions. Med Eng Phys.

[CR24] Plessers K, Mau H (2016). Stress analysis of a Burch-Schneider cage in an acetabular bone defect: A case study. Reconstr Rev.

[CR25] Ravera E, Crespo M, Guarnieri F, Braidot A (2015) Combined finite element and musculoskeletal models for analysis of pelvis throughout the gait cycle. In: Idelson SR, Sonzogni V, Coutinho A, Cruchaga M, Lew A, Cerrolaza M (eds) PANACM 2015. 1st Pan-American congress on computational mechanics and XI Argentine congress on computational mechanics. International Association for Computational Mechanics, Buenos Aires

[CR26] Sződy R, Kotormán I, Manó S, Csernátony Z, Bagi I, Borbás L, Hatos I (2017) Design and manufacturing of custom-made acetabular cages for the revison of hip joint implants: procedure applied in three cases. 7. In: Hungarian conference of biomechanics, Okt 6–7, Szeged, Hungary

[CR27] Totoribe K, Chosa E, Yamako G, Zhao X, Ouchi K, Hamada H, Deng G (2018). Acetabular reinforcement ring with additional hook improves stability in three-dimensional finite element analyses of dysplastic hip arthroplasty. J Orthop Surg Res.

[CR28] Vogel D, Klimek M, Saemann M, Bader R (2020). Influence of the acetabular cup material on the shell deformation and strain distribution in the adjacent bone—a finite element analysis. Materials.

[CR29] Wang G, Huang W, Song Q, Liang J (2017). Three-dimensional finite analysis of acetabular contact pressure and contact area during normal walking. Asian J Surg.

[CR30] Zaharie DT, Phillips ATM (2018). Pelvic Construct Prediction of Trabecular and Cortical Bone Structural Architecture. J Biomech Eng.

